# Depression Risk Factors for Knowledge Workers in the Post-Capitalist Society of Taiwan

**DOI:** 10.3390/healthcare10010137

**Published:** 2022-01-11

**Authors:** Hui-Li Lin, Fang-Suey Lin, Ling-Chen Liu, Wen-Hsin Liu

**Affiliations:** 1 Graduate School of Design, National Yunlin University of Science and Technology, Yunlin 64002, Taiwan; D10630018@yuntech.edu.tw (H.-L.L.); linfs@yuntech.edu.tw (F.-S.L.); 2 Department of Occupational Therapy, Asia University, Taichung 41354, Taiwan; 2000fawo@gmail.com; 3 Division of Family Medicine, Ditmanson Medical Foundation Chia-Yi Christian Hospital, Chiayi 600566, Taiwan

**Keywords:** post-capitalist society, knowledge worker, depression risk factor, individual characteristic

## Abstract

This study aimed to examine the depression risk factors for knowledge workers aged 20–64 in the post-capitalist society of Taiwan. Interview data from 2014 and 2019 were adopted for quantitative analysis of the depression risk by demographic and individual characteristics. The results showed that the depression risks of knowledge workers were not affected by demographic variables in a single period. From 2014 to 2019, the prevalence of high depression risk in knowledge workers aged 20–64 years increased over time. The more attention is paid to gender equality in society, the less the change in the gender depression index gap may be seen. Positive psychological state and family relationships are both depression risk factors and depression protective factors. Being male, married, religious, and aged 45–49 years old were found to be critical risk factors. Variables of individual characteristics could effectively predict depression risk.

## 1. Introduction

The 21st century has entered a post-capitalist society. Knowledge has replaced labor and capital as the most basic economic resource, making knowledge workers have gradually become the dominant players in society [1,2]. According to the statistical analysis from Directorate-General of Budget, Accounting and Statistics (DGBAS), Executive-Yuan, knowledge workers are mainly engaged in high-tech, information, and education industries in Taiwan [3]. They are the most important contributors to Taiwan’s economy and are highly valued by the government [4,5,6]. However, they must continue to learn and compete to avoid being eliminated. As a result, they become a high-risk group of depression under long-term high stress [7].

Depression in mental disorders is a common disease globally, affecting more than 264 million people [8]. Depression can cause individuals to suffer great suffering and perform poorly at work, school, and in the family. In the worst case, depression can lead to suicide [9]. The burden of disease caused by depressive disorders is calculated in terms of DALY. One DALY represents the loss of the equivalent of one year of full health [10]. The Global Burden of Disease (GBD) Study uses disability-adjusted life year (DALY) as an indicator to estimate the global disease burden and reveals that global disease burden estimates leading causes of DALYs from depressive disorders increased by more than 61% between 1990 and 2019, and depression is the main cause of non-fatal health loss for people aged 10–74, and the number of depression is gradually increasing [8]. From 2010 to 2019, laborers aged 20–64 accounted for 91% of the total international employed population. These people have made significant contributions and influences to global social and economic development [11,12,13,14].

People aged 20–64 years are the main contributors to the global economy, but they also comprise the high-risk group of depression [8,11,12,13,14]. In Taiwan, people aged 20–64 years constitute 96% of the employed population [15], which is higher than that in other regions of the world [11,12,13,14]. According to Taiwan’s official statistics, people aged 20–64 years were responsible for 75% of the medical expenses for mental disorders [16]. This indicates that, compared with other regions, the employed population in Taiwan aged 20–64 years has higher economic stress and depression risk. Although knowledge workers only account for 3.2% of the employed population aged 20 to 64 in Taiwan [17], they have to face high-pressure jobs for a long time. The environment makes their depression risk higher than other workers. This poses a latent crisis for Taiwan’s overall economy and social development, rendering depression prevention measures essential. Preventive plans can reduce depression risk [9] because they can prevent the development of etiological agents and diseases [18]. For controllable risk factors, preventive plans can be used to prevent or reduce depression risk. In epidemiology, risk refers to the probability of a healthy individual contracting a disease in the future. Risk factors refer to factors that increase the danger of contracting a disease [19]. Therefore, the risk factors for depression are relevant factors that may trigger depression symptoms or increase depression risk. Protective factors for depression are interference factors that can help people pass through stress periods and prevent depression development. Depression risks differ according to individuals’ physiological health, personal backgrounds, and personal characteristics [9].

Sex, age, marital status, and religious beliefs are critical factors for the appearance of mood disorders [20]. According to an epidemiology study in the United States, 2.2% of the population develops depression each month, and the probability of women developing depression is approximately twice as high as that of men [21]. In 2019, official statistics in Taiwan showed that the number of women with mood disorders is 1.73 times that of men [16], demonstrating that depression risk differs according to sex. Another study revealed a correlation between age and depression: Adults in the United States exhibited a U-shaped curve for physiological and psychological issues versus age [20]. By contrast, in Taiwan, adults demonstrated an upside-down U-shaped curve for mood disorders with age [13]. These results indicated that age and depression risk are correlated, but differences exist among different regions. Mead discovered that marital status is a critical impact factor for depression [22], compared with individuals who are married, those single or divorced have a significantly higher depression risk [23]. Studies have shown that religious beliefs can be a protective factor for depression. In general, it has a preventive function against depression morbidity [24], and the lower the participation in religious activities, the higher is the depression risk [25].

To reduce depression risk, positive psychology scholars have proposed the promotion of individuals’ positive mood experience and social–environmental interactions, all of which facilitate the formation of critical protective mechanisms to people prone to developing depression [26], providing individuals to have more opportunities to use their personal and environmental resources and relieve the stress they encounter [27]. However, people who are used to negative thoughts, have financial difficulties, or encounter setbacks in interpersonal relationships easily develop psychological stress due to negative life events, resulting in an increased depression risk [28].

The risk and protective factors for depression include physiological, psychological, and social aspects. Understanding the interactions between the protective and risk factors and interventions to alter their interactions are crucial to depression prevention. In epidemiology, diseases are believed to not occur randomly, rather due to certain factors. Describing these factors precisely is conducive to organizing a disease hypothesis. Analyzing the impact of the differences in demographic features on diseases’ incidence can provide further cues into the pathogenic mechanism of the diseases [29]. To prevent depression in knowledge workers in Taiwan from causing disabilities and disability-adjusted life year loss in individuals and losses to the whole society and economy, this study adopted a mixed perspective of epidemiology and psychology and approached the problem from the demographic and individual perspectives. This study’s research objectives are twofold.

On the basis of the results of questionnaires in 2014 and 2019, this study adopted a longitudinal approach, to investigate the impact factors of depression in knowledge workers aged 20–64 years in Taiwan. In addition, this study proposed risk and protective factors for depression so as to control the psychological health problems in knowledge workers in Taiwan.

Based on the aforementioned research objectives and literature review, this study proposed the following research questions:

(A) Do demographic characteristics affect the depression risk in knowledge workers?
(B) Do individual characteristics have depression risk predictive power in knowledge workers?
(C) Are there differences in the depression prevalence and OR from 2014 to 2019?

Subsequently, the following research hypotheses were proposed:

**Hypothesis** **1** **(H1).** *Sex, marital status, age, and religious beliefs affect depression risk*.

**Hypothesis** **2** **(H2).** *Positive psychological state, family relationship, and social class have an independent impact and predictive power on depression risk*.

Hypothesized regression model: Y_𝑖_ = β_0_ + β_1_*x*_1__𝑖_ + β_2_*x*_2__𝑖_ + β_3_ *x*_3__𝑖_ + ε_𝑖_ (𝑖 = 1, …, n):
β_1_: Positive psychological state regression coefficient;
β_2_: family relationship regression coefficient;
β_3_: social class regression coefficient;
H_0_: β_j_ = 0 (j = 1, 2, 3);
H_1_: β_j_ ≠ 0.

**Hypothesis** **3** **(H3).** *Depression prevalence and odds ratios (OR) in 2014 and 2019 differ significantly*.

Biological risk factors such as genetic and physiological diseases must be identified by medical experts, and analyzing these by using depression risk questionnaire results is difficult. Therefore, we did not include biological factors in this study.

## 2. Materials and Method

### 2.1. Study Design

**Figure 1:**
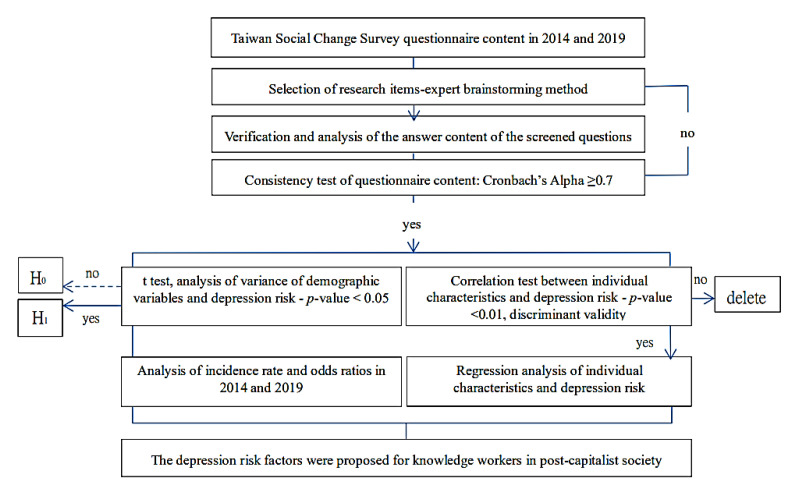
Study design.

To prevent personal experience and subjective evaluation from misguiding the understanding of the current depression risk and ensure the accuracy and reliability of the research results, this study adopted the questionnaire data from the official Taiwan Social Change Survey [30,31]. Based on the current objectives and research questions, we invited experts to screen through items in the 2014–2019 Taiwan Social Change Survey. Next, on the basis of the responses to the selected items, a longitudinal design was used. Through quantitative data analysis, we obtained the impact factors, prevalence, and ORs of depression risk in knowledge workers aged 20–64 years. The study design is shown in Figure 1.

### 2.2. Participants

The research participants were knowledge workers aged 20–64 years in the high-tech, information, and education industries in Taiwan.

### 2.3. Instrument

This study selected items from the 2014 and 2019 survey questionnaires. The instruments include structured problems such as demographic characteristics, individual characteristics, and depression risk. The demographic characteristics were sex, age, marital status, and religious belief, whereas the individual characteristics were a positive psychological state, family relationship, and social status. Moreover, medical experts were invited to discuss and determine positive psychological state items. The method to evaluate the “positive psychological state” is the questions of positive psychological state items, which were based on Derigatus SCL-90-R [32]. It has 4 questions that were scored on a 5-point scale ranging (0 point: very good; 1 point: good; 2 points: ordinary; 3 points: bad; 4 points: very bad). The principal component method was used to examine the questions, and one factor was selected and named “positive psychological state “. The characteristic value was greater than 1, and the explained variation was 65.46%. After verification, these items were found to achieve a Cronbach α of 0.8.

Regarding depression risk items, the samples were required to cover both patients with depression and control individuals. Medical experts were invited to discuss clinical diagnoses and norm control. Patient Health Questionnaire-9 (PHQ-9) [33] and 5-item Brief Symptom Rating Scale (BSRS-5) [34] are tools for evaluating depression risk. Their purpose is to enable rapid understanding of individuals’ needs for mental care, thereby revealing whether and which psychological health services are needed. We used PHQ-9 and BSRS-5 data to screen for the aforementioned items or depression risk. Considering that PHQ-9 and BSRS-5 data can achieve favorable reliability and validity among different patient groups, they can be applied broadly. The selected items were weighted using unequal selection probabilities and subjected to the internal consistency test. We found that their Cronbach α was 0.7.

Regarding the internal consistency and validity test results, depression and positive psychological state items (Cronbach’s α = 0.7 and 0.8, respectively) had internal consistency. Pearson’s correlation was conducted to individual characteristics and depression risk in 2019, and the *p* value < 0.01 was considered a significant correlation. The Cronbach’s α of each variable was greater than the correlation coefficient of any two variables, confirming discriminant validity.

### 2.4. Sampling and Data Sources

The data source in this study was the interview data from the 2014 and 2019 Taiwan Social Change Survey [30,31]. The survey adopts geographically stratified sampling and divided it into 19 layers. Stratified multistage probability proportional to size sampling was used for sample selection. Of the study population, every individual had an opportunity to be selected that was not 0. Each selected person could not be replaced by another. After receiving informed consent from the interviewees, interviewers conducted onsite interviews to obtain data. Pearson’s chi-squared test was applied to ensure that all the samples were representative. The investigation period was from 3 August 2014 to 16 November 2014 and from 29 July 2018 to 28 February 2019. The interview time for each participant was approximately 30 min to 45 min. In 2014 and 2019, after blank responses, those with unanswered questions, and those with missing answers were excluded, 182 and 180 valid responses remained, respectively.

### 2.5. Data Analysis

SPSS 16.0 [35] was employed for calculation and quantitative data analysis. *t* test and one-way analysis of variance were used for the correlation and difference analyses of the demographic variables in 2014 and 2019 with regard to depression risk. In the multiple regression analysis, the F test, *t* test, and variance inflation factor (VIF) were used to assess the predictive power of individual characteristics for depression risk.

To objectively assess risk factors for depression, we compared depression prevalence and ORs among interviewees aged 20–64 years in 2014 and 2019 and performed a comprehensive assessment with regression analysis, thereby proposing risk and protective factors for depression.

## 3. Results

### 3.1. Demographic Characteristics Analysis

The frequency distribution of the demographic characteristics of the knowledge workers aged 20–64 years in the 2014 and 2019 surveys showed that most participants were married, and >80% of people were religious (Table 1), which is in line with the fact that Taiwan has diverse and blooming religions.

The participants were divided into nine groups based on their age. In 2014, the risk mean of each age group was between 1.66 and 2.01, with SE between 0.090 and 0.137. The risk means in the age groups 35–39 and 50–64 were lower than the total risk mean. The risk means of the age groups 60–64 and 25–29 were 1.67 and 2.01, respectively, which were the lowest and highest, respectively. In 2019, the risk mean of each age group was between 2.16 and 2.75, with SE between 0.050 and 0.198. The risk means in the age groups 25–34 and 55–64 were lower than the total risk mean. The risk means of the age groups 60–64 and 50–54 were 2.17 and 2.72, respectively, which were the lowest and highest, respectively.

### 3.2. Depression Risk Differences

#### 3.2.1. Independent Samples *t* Test for the Effects of Sex, Marital status, and Religious Beliefs on Depression Risk

Independent samples *t* test was used to analyze the differences in the effects of sex, marital status, and religious beliefs on depression risk in 2014 and 2019, as presented in Table 2.

As the *p*-Value in the F test for sex, married status, and religion was >0.05, we selected the result “assuming all variables are equal” in the *t* test in 2014 and 2019, and therefore, the *p*-Value of *t* test was >0.05 in the two periods. Thus, depression risks in sex, married status, and religion did not differ significantly.

#### 3.2.2. One-Way Analysis of Variance for the Effects of Age on Depression Risk

Levene’s test of equal variance was performed for the effect of age on depression risk in each group. In 2014, Levene’s statistic was found to be 1.72, with the degrees of freedom of the numerator and denominator = 8 and 173, respectively, and *p*-Value = 0.097, indicating Homogeneity, F test statistic was found to be 0.646 and *p*-Value = 0.738. In 2019, Levene’s statistic was found to be 1.523, with the degrees of freedom of the numerator and denominator = 8 and 171, respectively, and *p*-Value = 0.152, indicating homogeneity. F test statistic was found to be 1.011, and *p*-Value = 0.429. Therefore, it was impossible to reject the null hypothesis that the averages of each group were equal. No significant differences were observed in the depression risk among age groups in 2014 and 2019.

### 3.3. Multiple Regression Analysis for the Effects of Individual Characteristics on Depression Risk

By using the F test, *t* test, and VIF in the regression model, we performed prediction analysis of individual characteristics for depression risk (Table 3 and Table 4).

The regression model in 2014 derived F = 0.012 ((*p*-Value = 0.912; Table 3); therefore, H_0_ was not rejected, indicating that the regression model had not predictive power. The regression model in 2019 derived F = 28.185 ((*p*-Value = 0.000; Table 3); therefore, H_0_ was rejected—indicating that the regression model had predictive power.

The *t* test of individual regression coefficients showed the following: *t* for positive psychological state (*x*_1_), family relationship (*x*_2_), and social status (*x*_3_) was −5.517 (*p*-Value = 0.00 < 0.05; H_0_ was rejected), −4.837 (*p*-Value = 0.00 < 0.05; H_0_ was rejected), and −0.622 (*p*-Value = 0.535 > 0.05; H_0_ was accept). In other words, positive psychological state (*x*_1_) and family relationship (*x*_2_) had significant influence on depression risk (y), whereas social status (*x*_3_) had not a significant influence on depression risk (y).

**Figure 2:**
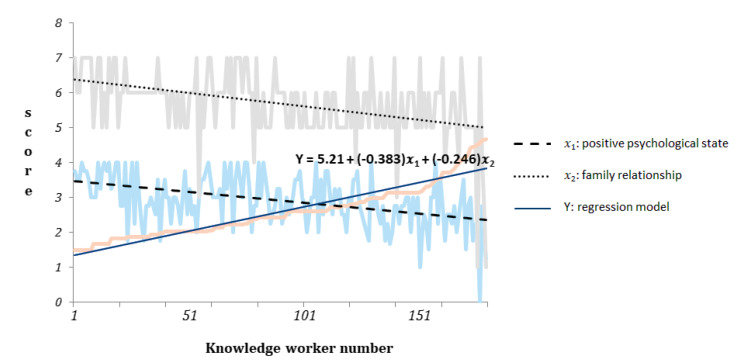
Graphs of regression programs for depression-individual characteristics.

Moreover, their VIF values were 1.081, 1.075, and 1.050, respectively, (all <10), confirming that their collinearity was not prominent. Finally, the regression model Y = 5.21 + (−0.383) (*x*_1_) + (−0.246) (*x*_2_) could predict depression risk effectively (Figure 2).

### 3.4. Longitudinal Analysis of Depression Risk Prevalence and Odds Ratio (OR)

To determine the correlation between depression risk factors and its risk without errors, we used absolute measurements [29]. To determine the risk factors for depression risk precisely, this study analyzed the prevalence and OR of the demographic variables. Prevalence refers to the ratio of the number of people developing a disease among a population in a certain amount of time (prevalence = existing cases in a group in a certain amount of time/total population of the group) [36]. OR is an indicator showing the relationship between risk factors and disease occurrence (OR = the ratio of exposure in the case group/the ratio of exposure in the control group) [36].

We considered the responses to every dimension in the demographic variables. People who scored higher than the mean score in the depression risk items were considered the high depression risk group. By contrast, people who scored lower than the mean score in the depression risk items were considered the control group.

Moreover, the groups with higher proportions of depression risk were considered the exposure group. It comprised men among the sex group, those who were married among the marital status group, those aged 45–49, 55–59 years among the age group, and people with religious beliefs among the religious belief group. All the remaining groups were considered non-exposure groups. The prevalence and OR analysis results for the depression risk are shown in Table 5.

From 2014 to 2019, depression risk prevalence increased in each group except for the group aged 55–59 years old. It increased the most in those aged 45–49 (from 46.43 to 76.47), followed by the male group. OR increased in the sex and 45–49-year-old groups but decreased in the other three groups. In 2014 and 2019, high depression risk was 1.08 and 1.16 times as common in women than in men, respectively. The correlation between males and depression risk exhibited a greater correlation over time. In 2014, the OR of the age group 55–59 was > 1, whereas it was 0.92 times that of other age groups in 2019. Thus, the correlation between the group aged 55–59 years and depression risk also exhibited differences over time.

## 4. Discussion

This study showed that the risks of depression by knowledge workers were not affected by demographic variables in various periods. In general, from 2014 to 2019, the prevalence of high depression risk increased, possibly indicating that the surveyed people were exposed to an environment with more risk factors. Therefore, depression prevention has become increasingly crucial. The current ORs data indicated specific high-risk factors that can be considered in prevention plans. In the current longitudinal study, we discovered that the ORs of demographic variables changed over time. For instance, the age range of 55–59 years changed from being a risk factor to being a protective factor. In terms of multiples of ORs, marriage and religion decreased, while sex and 45–49 age groups increased. The results showed that people with certain demographic characteristics were affected by the change of time, gradually having increased depression risk. The study results showed that male ORs were 1.08 (2014) and 1.16 (2019) times higher than female ORs. The risk of depression of knowledge workers is higher in men than in women. In Taiwan, women receive more education than men, and their income is gradually becoming closer to that of men [37,38]. Consequently, men are competing with women for the same positions at work and sharing familial responsibilities; these may be critical factors increasing depression risk among men. The National Institute of Mental Health reported that some men with depression hide their emotions and are less likely to identify with, discuss, and seek help for depression, compared with women. In addition, men who are depressed may appear to be angry or aggressive instead of sad [39]. Men commonly use alcohol and other substances to self-treat depression, which only exacerbates their mental health problems and increases their risk of other health problems [39,40]. Relevant statistics and the current results have shown the probability of depression risk in male knowledge workers being underestimated. The psychological health of male knowledge workers has become a public health problem worthy of attention [41]. Many studies in the literature reveal the main reason for women being more melancholy than men is that women’s low socioeconomic status makes them prone to face more situations in life, causing them to be more melancholy than men [42,43,44,45,46]. In addition, under traditional patriarchy, women lack autonomy [47]. The depression of gender stereotypes and gender role-playing, housework, parenting, marriage problems, and long widowhood years can easily cause women to bear more pain and anxiety than men [48,49,50]. It also makes women’s depression index higher [51]. However, some studies have shown that countries that value gender equality, such as Denmark [52], Greece [46], Japan [45,53], and the United States [54], have very small gaps in gender depression indices. Countries in which there is no clear difference in gender depression index include South Korea [43,44,55], Canada [56], etc. According to the calculation method of Human Development Indices and Indicators 2018 Statistical update (UNDP), calculating the gender inequality index (GII) of Taiwan in 2018 shows that Taiwan’s GII is 0.045, which is lower than other regions in Asia, and is also the sixth-lowest score in the world [57]. This shows that Taiwanese society places great importance on gender equality, and it may also be an important difference between the results of this study and many previous studies in the literature that use women as risk factors for depression. This research result represents that Taiwan and other regions can improve certain parts of the depression risk factors by improving the problem of gender inequality, bringing about improvement of people’s health, and promoting the positive development of society.

The ORs of the 44–49 age group were 1.20 (2014) and 1.67 (2019) times the ORs of other age groups, and the risks of depression of the age 44–49 group were higher than other age groups. Those aged 44–49 years are more worried about unemployment, economics, social security, and other issues than other age groups. Meanwhile, they have the responsibility to take care of children and parents, which puts them at a higher risk of depression than other groups [58]. From 2014 to 2019, the ORs of marriage and religion declined, but the ORs of the married group and the faith group were consistently higher than those of the control group. These reflected that males and the 44–49 age group were key risk factors for depression. In addition, the married group and the belief group were also risk factors for depression. The ORs of married and religious beliefs were greater than those of the control group. Related research shows that the dual pressure of work and family is an important predictor of the mental and physical health of Taiwanese workers [59]. In the study of working stress, the dual roles of work and family cause greater pressure on individual knowledge workers [60]. This indicates that the stressor of married people was work as well as family, which leads to a higher risk of depression for married knowledge workers. In addition, the researchers conducted open-end interviews with five knowledge workers on the issue of religious beliefs, and the preliminary conclusion is that religious beliefs may be a channel for the high-risk group to seek stress relief, leading to higher depression risk in these two groups. This shows that religious belief may be one of the ways to relieve the pressure of knowledge workers. These observations need to be investigated in the future.

A positive psychological state and familial relationship can reduce depression risk. The social status had no effect on the depression risk. Thus, incorporating positive psychological state and familial relationship into the depression prevention plans for knowledge workers aged 20–64 years may result in protective effects. Through the 2014 and 2019 surveys, we found that the risk and protective factors for depression in knowledge workers changed as society changed.

The results of this study show that there are differences between knowledge workers and the general public in the depression risk factors, which are different from many studies in the literature. It also reveals that the knowledge workers with high depression risk are ignored, which can cause effective prevention and treatment measures not to be taken for high-risk knowledge workers. As risk factors must be closely related to disease to serve as valuable screening items, unnecessary risk factors should be removed from screening tests. The results of this study proposed that risk factors were closely related to depression for knowledge workers and can be used as a screening item for depression. Based on the findings of this research, mental health professionals and policy providers may improve related strategies with time by understanding relevant impact factors for depression, as well as by establishing related plans and providing more favorable support to face risk factors for mental health problems.

## 5. Conclusions

The long-term trend showed that the depression risk for knowledge workers in the post-capitalist society of Taiwan had gradually increased over time. Therefore, depression prevention has become increasingly crucial. Male, married, religious belief, and 44–49 age groups were high risk factors of depression. Male knowledge workers aged 45–49 years were the highest risk group. However, the more attention is paid to gender equality in society, the less change in the gender depression index gap may be seen. Individual characteristics can effectively predict depression risk. The more positive the psychological state and familial relationship are, the lower is the depression risk, and vice versa. Positive psychology and familial relationships are the main depression risk factors for knowledge workers in the post-capitalist society of Taiwan, and they are also protective factors.

## Figures and Tables

**Table 1:** Demographic characteristics statistics of knowledge workers.

Demographic Characteristics		2014 (*n* = 182, Mean = 1.88, Std. Error = 0.04)		2019 (*n* = 180, Mean = 2.58., Std. Error = 0.06)	
		Number (%)	Mean (SE)	Number (%)	Mean (SE)
Sex	Male	79 (43.4)	1.91 (0.06)	64 (36.0)	2.73 (0.09)
	Female	103 (56.6)	1.87 (0.05)	116 (64.0)	2.50 (0.07)
Age (years)	20–24	21 (11.5)	1.92 (0.13)	22 (12.2)	2.68 (0.17)
	25–29	27 (14.8)	2.01 (0.11)	25 (13.9)	2.54 (0.11)
	30–34	19 (10.4)	1.86 (0.12)	18 (10.0)	2.45 (0.16)
	35–39	21 (11.5)	1.83 (0.09)	26 (14.4)	2.74 (0.16)
	40–44	19 (10.4)	1.90 (0.09)	14 (7.8)	2.63 (0.24)
	45–49	28 (15.4)	1.96 (0.12)	17 (9.4)	2.66 (0.18)
	50–54	17 (9.3)	1.80 (0.14)	28 (15.6)	2.72 (0.16)
	55–59	16 (8.8)	1.86 (0.12)	19 (10.6)	2.38 (0.19)
	60–64	14 (7.7)	1.67 (0.11)	11 (6.1)	2.17 (0.11)
Marital status	single	86 (47.0)	1.83 (0.06)	94 (52.2)	2.54 (0.07)
	married	96 (53.0)	1.93 (0.05)	86 (11.0)	2.63 (0.08)
Religious belief	Yes	152 (84)	1.89 (0.04)	149 (82.8)	2.57 (0.14)
	no	29 (16)	1.87 (0.10)	31 (17.2)	2.63 (0.06)

Mean: mean of depression risk.

**Table 2:** F test and *t* test of depression risk in sex, marital status, and religious beliefs.

Levene’s Test					*t* Test			
Demographic Characteristics	F Test	Sig.	*t*	*df*	*p*-Value	Difference between Means	SE of Difference	95% CI
Sex								
2014	0.996	0.32	0.529	180	0.596	0.41	0.08	−0.113–0.196
2019	0.005	0.95	1.91	178	0.058	0.22	0.116	−0.007–0.452
Married status								
2014	0.490	0.48	1.304	180	0.194	−0.101	0.08	−0.254–0.052
2019	0.180	0.67	0.857	178	0.393	−0.10	0.11	−0.319–0.126
Religious beliefs								
2014	0.08	0.77	0.23	179	0.815	0.02	0.10	−0.185–0.234
2019	0.008	0.93	0.383	178	0.702	0.06	0.15	−0.240–0.350

Mean: mean of depression risk.

**Table 3:** Analysis of variance ^b^ in 2014 and 2019.

Model 1	Sum of Squares	*df*	Mean Square	F	Sig.
2014 Regression	0.003	1	0.003	0.012	0.912
Residual	49.465	180	0.275		
Total	49.469	181			
2019 Regression	33.011	3	11.004	28.185	0.000 ^a^
Residual	68.712	176	0.390		
Total	101.723	179			

^a^ predictors (constant): positive psychological state (2019), family relationship (2014, 2019), and social class (2019); ^b^ dependent variable: depression risk.

**Table 4:** Collinearity diagnostics of regression coefficient ^a^ in 2019.

		Unstandardized Coefficients		Standardization Factor	*t*	Sig.	Collinearity Diagnostics	
Model		B	Std. Error	Beta			Tolerance	VIF
1	(constant)	5.21	0.318		16.365	0.000		
Positive psychological state		−0.383	0.069	−0.364	−5.517	0.000 *	0.881	1.135
Family relationship		−0.246	0.051	−0.321	−4.837	0.000 *	0.869	1.150
social class		−0.019	0.031	−0.039	−0.622	0.535	0.963	1.038

^a^ dependent variable: depression risk. * model shows significant influence on depression risk.

**Table 5:** Prevalence and ORs in the group with a high risk of depression.

Demographic Characteristic		Prevalence (Number of High-Risk Persons (Group)/Total Number of Effective Samples in Group)		ORs (Exposure Ratio of High-Risk Group/Exposure Ratio of Control Group)	
		2014	2019	2014	2019
Sex	A: male	43.04	53.13	1.08	1.16
	B: female	39.81	45.69		
Age (years)	A: 45–49	46.43	76.47	1.20	1.67
	B: other age groups	38.85	45.40		
	A: 55–59	50.00	42.11	1.29	0.92
	B: other age groups	38.85	45.40		
Marital status	A: married	43.75	51.16	1.14	1.12
	B: single	38.37	45.74		
Religious belief	A: yes	43.42	50.34	1.40	1.30
	B: no	31.03	38.71		

A: exposure group; B: control group.

## Data Availability

The original data of this study are available from Survey Research Data Archive, Academia Sinica by application.
